# Selective targeting of the oncogenic *KRAS* G12S mutant allele by CRISPR/Cas9 induces efficient tumor regression

**DOI:** 10.7150/thno.42325

**Published:** 2020-04-06

**Authors:** Qianqian Gao, Wenjie Ouyang, Bin Kang, Xu Han, Ying Xiong, Renpeng Ding, Yijian Li, Fei Wang, Lei Huang, Lei Chen, Dan Wang, Xuan Dong, Zhao Zhang, Yanshan Li, Baichen Ze, Yong Hou, Huanming Yang, Yuanyuan Ma, Ying Gu, Cheng-Chi Chao

**Affiliations:** 1 Guangdong Provincial Key Laboratory of Genome Read and Write, BGI-Shenzhen, Shenzhen 518083, China; 2 China National GeneBank, BGI-Shenzhen, Jinsha Road, Shenzhen 518120, China; 3 Guangdong Provincial Academician Workstation of BGI Synthetic Genomics, BGI-Shenzhen, Shenzhen, 518083, China; 4 Department of Thoracic Surgery II, Key Laboratory of Carcinogenesis and Translational Research (Ministry of Education/Beijing), Peking University Cancer Hospital and Institute, Beijing, China; 5 BGI Education Center, University of Chinese Academy of Sciences, Shenzhen, 518083, China; 6 James D. Watson Institute of Genome Sciences, Hangzhou 310058, China; 7 Ab Vision, Inc, Milpitas, California, USA

**Keywords:** * KRAS* mutation, CRISPR/Cas9, dCas9-KRAB, mRNA-regulating, cancer therapy

## Abstract

**Rationale**: *KRAS* is one of the most frequently mutated oncogenes in cancers. The protein's picomolar affinity for GTP/GDP and smooth protein structure resulting in the absence of known allosteric regulatory sites makes its genomic-level activating mutations a difficult but attractive target.

**Methods**: Two CRISPR systems, genome-editing CRISPR/SpCas9 and transcription-regulating dCas9-KRAB, were developed to deplete the *KRAS* G12S mutant allele or repress its transcription, respectively, with the goal of treating *KRAS*-driven cancers.

**Results**: SpCas9 and dCas9-KRAB systems with a sgRNA targeting the mutant allele blocked the expression of the mutant *KRAS* gene, leading to an inhibition of cancer cell proliferation. Local adenoviral injections using SpCas9 and dCas9-KRAB systems suppressed tumor growth *in vivo*. The gene-depletion system (SpCas9) performed more effectively than the transcription-suppressing system (dCas9-KRAB) on tumor inhibition. Application of both Cas9 systems to wild-type *KRAS* tumors did not affect cell proliferation. Furthermore, through bioinformatic analysis of 31555 SNP mutations of the top 20 cancer driver genes, the data showed that our mutant-specific editing strategy could be extended to a reference list of oncogenic mutations with high editing potentials. This pipeline could be applied to analyze the distribution of PAM sequences and survey the best alternative targets for gene editing.

**Conclusion**: We successfully developed both gene-depletion and transcription-suppressing systems to specifically target an oncogenic *KRAS* mutant allele that led to significant tumor regression. These findings show the potential of CRISPR-based strategies for the treatment of tumors with driver gene mutations.

## Introduction

A high frequency of *RAS* mutations has been found in various types of human cancers, including colon 1, 2, lung 3, and pancreatic 4 cancers, which are the most deadly malignancies worldwide 5. The three *RAS* oncogenes, *NRAS*, *HRAS*, and *KRAS*, make up the most frequently mutated gene family in human cancers. *KRAS* mutations are the most prevalent (21%) among the three genes, while the other two mutations are 3% and 8% for *NRAS* and *HRAS*, respectively 6.

*KRAS* is predominantly mutated in pancreatic ductal adenocarcinomas (PDACs), colorectal adenocarcinomas (CRCs), and lung adenocarcinomas (LACs) 7. A majority of oncogenic *KRAS* mutations occur at codon 12, 13, and 61. G12 mutations are the most common variations (83%). It was reported that *KRAS* G12S is present in 1.84% of all colorectal adenocarcinoma patients, while only present in 0.5% of non-small cell lung carcinoma patients 8 (Table 1).

Based on the well-validated role of mutation- induced activation of KRAS in driving cancer development and growth, comprehensive efforts have been undertaken to develop therapeutic strategies to halt mutant KRAS function for cancer treatment. Different strategies to inhibit KRAS signaling have been under investigation, including exploring direct KRAS-binding molecules, targeting proteins that facilitate KRAS membrane-associated or downstream signaling, searching for synthetic lethal interactors and novel ways of inhibiting *KRAS* gene expression, and harnessing the immune system 9-11. While KRAS G12C inhibitors are now in early phase clinical trials with encouraging results (NCT03600883, NCT03785249) 12, 13, the past three decades of KRAS-targeted therapy had not shown a significant clinical benefit.

The numerous studies involving blocking the RAS pathway have demonstrated the necessity to pursue mutation-specific RAS-targeted strategies. Small molecules that selectively bind to the KRAS G12C mutant were reported but demonstrated limited effects *in vitro* 14. Gray et al. also targeted KRAS-G12C with a GDP analogue which could covalently bind to the cysteine of the G12C mutant, but was limited by its ability to penetrate into cells 15. Synthetic lethal interactors have also been screened in G13D 16, 17 or Q61K 18 mutant cell lines to specifically target cancer cells, but are still far out from clinical applications. Despite the various attempts to directly interfere with KRAS, this protein is still considered to be a challenging drug target due to the lack of a suitable binding pocket for small molecule inhibitors in its structure 10.

Development of antibodies and small molecule inhibitors is cost-ineffective and time consuming. Compared to the traditional antibody or inhibitor which is mainly used to alter one specific target, genome editing technology could be a better alternative to flexibly manipulate biological activity of designated molecules at the DNA level. The CRISPR/ SpCas9 system, developed from *Streptococcus pyogenes*, recognizes specific DNA sequences and is widely applied to the genome editing of mammalian cells 19, 20. Taeyoung Koo et al. has used CRISPR/ Cas9 to target an epidermal growth factor receptor (*EGFR*) oncogene harboring a single-nucleotide missense mutation to enhance cancer cell killing 21. Zhang-Hui Chen et al. targeted genomic rearrangements in tumor cells through insertion of a suicide gene by Cas9 22. Those findings have preliminarily proved the concept of specifically disrupting mutant tumors by using the CRISPR/Cas9 system. *KRAS* mutant alleles, including G12V, G12D, and G13D, have also been targeted by the CRISPR/Cas9 system to control tumor growth 23, 24. In addition, the CRISPR-Cas13a system was engineered for the targeted therapy of *KRAS*-G12D and *KRAS*-G12C mutants in pancreatic cancer 25. Although the above mentioned three *KRAS* mutant alleles have become established targets for the CRISPR/Cas9 genome- editing system, the G12S mutation, with rectal adenocarcinoma, colorectal adenocarcinoma, and colorectal carcinoma having greater prevalence than the other cancer types (Table 1) 26, has not yet been targeted by the CRISPR system.

Here we demonstrate that the G12S mutant allele can be specifically targeted by the CRISPR/SpCas9 system, while leaving the wild-type *KRAS* allele unaffected. The delivery of SpCas9 and a guide RNA targeting the G12S mutant allele affected the *in vitro* proliferative ability and cell cycle of tumor cells, and the *in vivo* tumor growth. Besides the genome-editing CRISPR/Cas9 system, a transcription-regulating dCas9-KRAB system 27, which binds to the target sequence using dCas9 and downregulates mRNA transcription using the transcriptional repressor KRAB, was also applied to inhibit tumor growth. However, the dCas9-KRAB system was less effective than the CRISPR/Cas9 genome-editing system. Furthermore, the specific CRISPR targeting sites of 31555 oncogenic mutations in the top 20 cancer driver genes were screened using our high-throughput bioinformatics analysis, which allows for the application of this genome editing strategy to other cancer mutations. Our study is the first to target the *KRAS*-G12S mutant with the CRISPR/Cas9 and dCas9-KRAB systems for inhibition of tumor growth. The bioinformatic pipeline for analyzing the distribution of protospacer adjacent motif (PAM) sequences could also be a useful tool for the screening of edited targets. Combining next generation sequencing (NGS) with the genome-editing approach would be a promising strategy for targeting *KRAS* or other oncogenic mutations for personalized cancer treatment.

## Results

### Cas9-sgG12S specifically targeted KRAS mutant alleles

**Figure 1:**
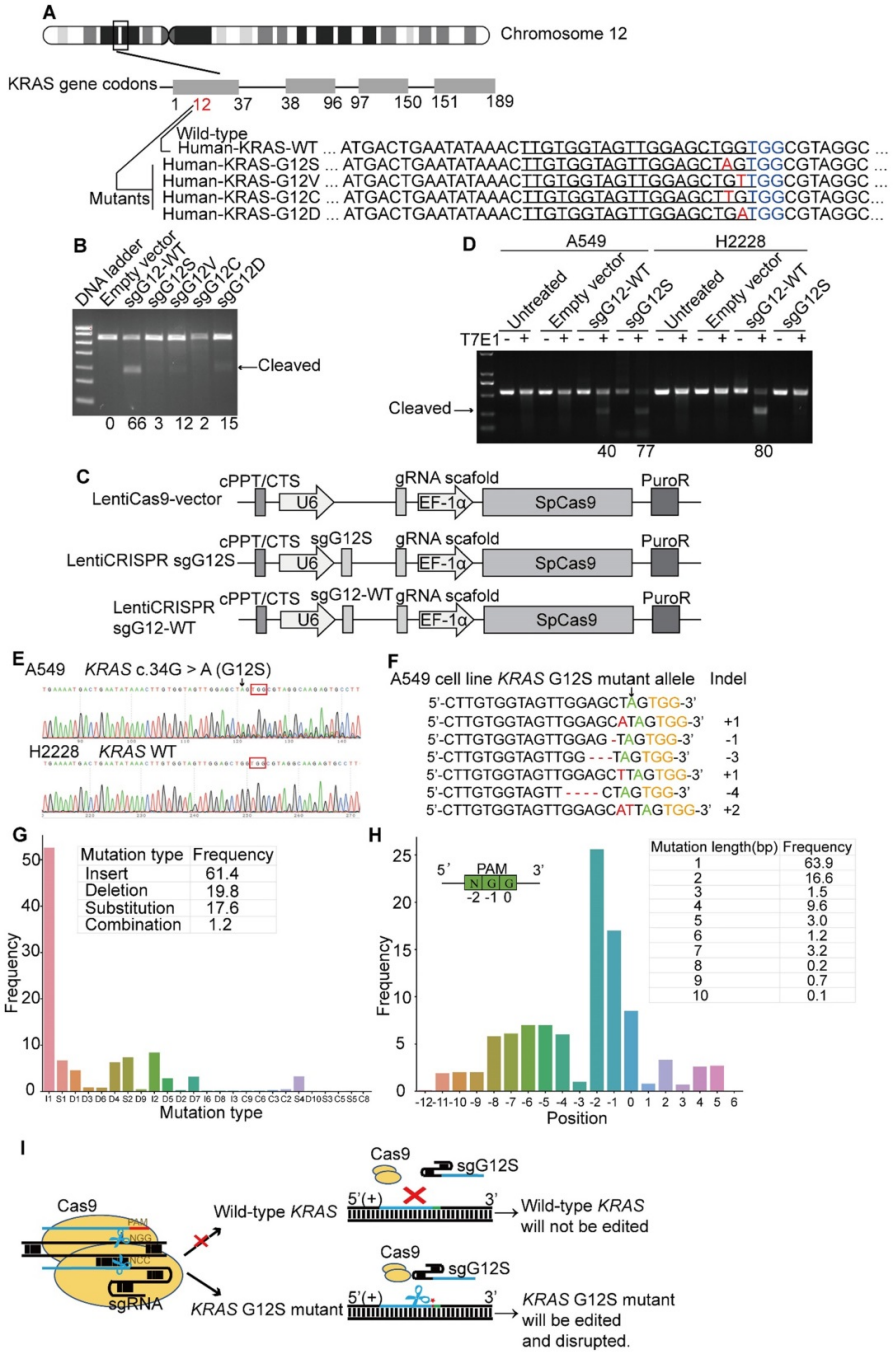
*KRAS* G12S oncogenic mutant-specific Cas9. (**A**) Mutations (red) at *KRAS* G12 site located in the seed sequence of a PAM (blue). The human *KRAS* gene is located on chromosome 12. Oncogenic single-nucleotide substitutions within exon-1 of *KRAS* (c. 34G>A, c.35 G > T, c.34 G>T and c.35 G > A) result in G12S, G12V, G12C, and G12D mutations. Sequences of their corresponding gRNAs are underlined. (**B**) Editing efficiency of different gRNAs in 293T cells. Effective editing of genes is presented by the appearance of a cleaved band. The gene editing efficiency is listed at the bottom of the corresponding lanes. (**C**) Maps of lentiviral constructs, including the LentiCas9-vector, sgG12S, and WT guide RNA expressing vectors. (**D**) Efficiency and specificity of sgG12S and sgG12-WT in A549 and H2228 tumor cells infected with sgG12S or sgG12-WT lentiviruses 48 h post-infection. Untreated and empty vector-infected cells served as controls. Effective editing of genes is presented by the appearance of a cleaved band. The gene editing efficiency is listed at the bottom of the corresponding lanes. (**E**) Gene editing event was confirmed by sanger sequencing in A549 and H2228 cells. The PAM sequence is marked by a red box and the *KRAS* G12S mutant allele is pointed out by a black arrow. (**F**) Mismatched nucleotides are shown in red, the *KRAS* G12S mutant allele in green pointed out by a black arrow, and PAM sequences in yellow. The right column indicates the number of inserted or deleted bases. (**G**) Mutation type and frequencies at the *KRAS* G12S site targeted by CRISPR/Cas9. I, insertion; D, deletion; S, substitution; C, combination. (**H**) Mutation positions, lengths, and frequencies at the *KRAS* G12S site targeted by CRISPR/Cas9. Sequence direction is shown in the top right. (**I**) Diagram of the genome therapy strategy to specifically target the *KRAS* G12S mutant allele. Blue strands: spacer; green strands: PAM sequence; red strands and star: single-nucleotide missense mutations.

The* KRAS* gene is located in the short arm of human chromosome 12. There are four dominant mutant alleles at the G12 position in exon 1, G12S (c.34G>A), G12V (c.35G>T), G12C (c.34G>T), and G12D (c.35G>A) (Figure 1A). These single nucleotide missense mutations are next to a PAM (TGG) sequence recognized by SpCas9. Since variations of DNA bases in the PAM or seed sequences can affect the recognition of SpCas9, five sgRNAs in total were designed to target the four *KRAS* mutant alleles, including G12S (sgG12S), G12V (sgG12V), G12C (sgG12C), and G12D (sgG12D), and the *KRAS*-WT gene (single guide G12 wild-type RNA, sgG12-WT).

We first examined the activity of these five sgRNAs in 293T cells (Figure 1B), which harbors the wild-type *KRAS* gene. To confirm the editing efficiency of sgG12-WT, and the specificity of sgG12- Mu (mutant), we transfected plasmids encoding spCas9 and different sgRNAs (Supplementary Figure S1A) into 293T cells separately. We found that the sgG12-WT disrupted *KRAS*-WT effectively with an efficiency of 66% by a T7E1 assay, while the editing efficiency of sgG12S, sgG12V, sgG12C, and sgG12D in *KRAS*-WT were 3%, 12%, 2%, and 15%, respectively (Figure 1B). Thus, sgG12S and sgG12C were more specific with much lower off-target effects on wild-type *KRAS*. Next, we confirmed the editing efficiency of sgG12S in A549 lung adenocarcinoma cells harboring the *KRAS* G12S mutant allele. H2228, another lung adenocarcinoma cell line carrying no G12S mutant allele, was utilized as a negative control. A549 and H2228 cells were infected by lentivirus containing spCas9-sgG12S or spCas9-sgG12-WT and a non-targeting control virus (Figure 1C), respectively. We found that the spCas9-sgG12S was able to edit the *KRAS* G12S mutant allele in A549 cells with a high efficiency of 77%, but there was limited or no editing efficiency in the wild-type *KRAS* allele in H2228 cells (Figure 1D). On the other hand, sgG12-WT was able to edit *KRAS* in A549 and H2228 cells with an editing efficiency of 40% and 80%, respectively, indicating that the sgG12-WT non-specifically bound to the *KRAS* G12S sites with a high mismatch tolerance. To further confirm that the sgG12S specifically edited the *KRAS* G12S mutant allele, but not the wild-type allele, the *KRAS* gene in puromycin-selected A549 and H2228 cells was sequenced 2-3 days post infection (Figure 1E). Next generation sequencing (NGS) showed that indels occurred in the *KRAS* G12S allele edited by spCas9-sgG12S (Figure 1F). Among all the generated mutations, insertions (61.4%) and deletions (19.8%) occupied most of the mutations, rather than substitutions (17.6%) and combinations (1.2%, ≥2 types of edition). In addition, 1 bp insertions (I1, 52%) occurred more frequently than other types, eg. I2, S2, et al. (Figure 1G). The mutated positions were analyzed to further explore where the mutations occurred, and most mutations (26%) occurred at the N of the NGG PAM sequence. Most mutations were changed by 1 bp (63.9%), followed by a 2 bp change (16.6%). Overall, based on bioinformatic analysis, most mutations were less than a 10 bp change at the *KRAS* G12S locus (Figure 1H). To summarize, *KRAS* in A549 was destroyed around the PAM (TGG) sequence, while H2228 was not affected, further confirming the success of our spCas9-sgG12S system in efficient and specific targeting of the *KRAS* G12S allele (Figure 1F).

### Genome editing of the KRAS G12S mutant allele inhibited the proliferation and cell cycle of tumor cell lines *in vitro*

**Figure 2:**
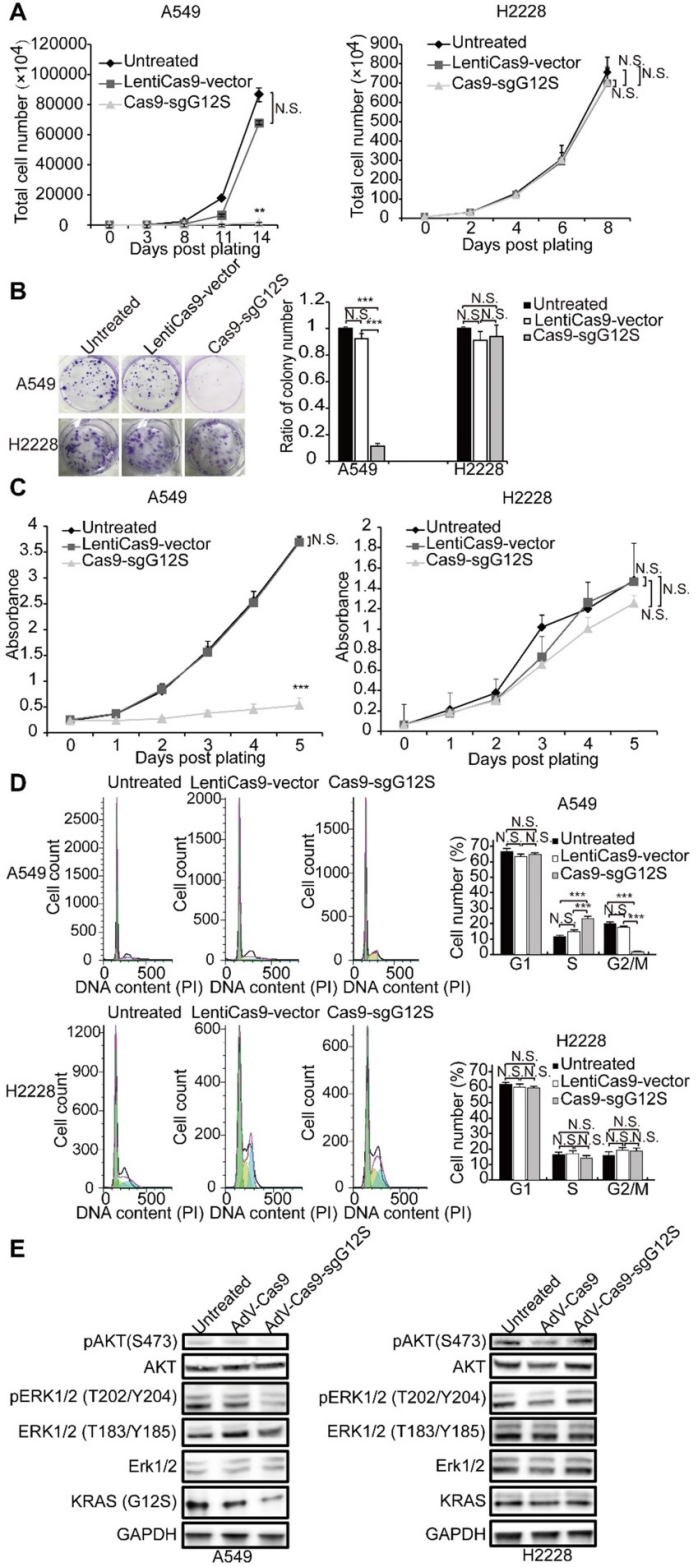
The anti-tumor effects of targeting the *KRAS* G12S mutant allele *in vitro*. A549 and H2228 cells were subjected to cell proliferation (**A**), colony forming (**B**), CCK-8 (**C**), cell cycle (**D**), and WB (**E**) assays after treatment with lentiviral Cas9 and sgRNAs targeting the *KRAS* G12S mutant allele. Error bars represent S.E.M. (∗) 0.01<P < 0.05, (∗∗) 0.001<P < 0.01, (∗∗∗) P < 0.001. (**A**) Cell growth curves were determined by counting cell numbers with various treatments at different timepoints. (**B**) Colony formation assay in A549 and H2228 cells. Representative images of wells after 0.5% crystal violet staining are shown at left. Colony number was determined 2 weeks after cell plating and treatment with LentiCas9-vector and Cas9-sgG12S. (**C**) CCK-8 assay in A549 and H2228 cells. Cell proliferation was determined using CCK-8 reagents at different timepoints after plating. The number of cells in cultures with different treatments was accessed by the optical density at 490 nm of each CCK-8 reaction. (**D**) Cell cycle was determined by PI staining and FACS analysis. (**E**) Western blot analysis of the phosphorylation levels of AKT and ERK proteins.

To investigate whether targeting and disruption of the *KRAS* mutant allele by sgG12S could inhibit the proliferation of tumor cells, the cell numbers of A549 and H2228 cells were examined after gene editing (Figure 2A). The proliferation of sgG12S-targeted A549 cells was dramatically inhibited and almost retarded compared to the non-targeting control and untreated groups. Meanwhile, the targeting of sgG12S had no effect on the proliferation of H2228 cells. In addition, a cell colony formation assay (CFA) (Figure 2B) and CCK-8 cell proliferation assay (Figure 2C) confirmed the growth inhibition by Cas9- sgG12S targeting. As demonstrated by cell counting (Figure 2A), the proliferation of A549 cells was significantly suppressed, shown in the CFA and CCK-8 assays. In contrast, the targeting of sgG12S had a lesser effect on the proliferation of H2228 cells carrying the wild-type *KRAS* allele. To exclude the possibility that random DNA breaks induced by Cas9, rather than specific disruption of mutant *KRAS* allele, are contributing to the specific inhibition of tumor cell proliferation, a CCK-8 cell proliferation assay was performed after treatments with Cas9 and control sgRNAs targeting *AAVS1* (the adeno-associated virus integration site 1) and *TTN* genes. The *AAVS1* locus in the first intron of the *PPP1R12C* gene is one of the most commonly used genomic safe harbor (GSH) sites in human cell research 28, and TITIN encoded by *TTN* gene is one of the myofibrillar proteins thought to play an important role in the assembly and function of muscle sarcomeres 29. Disruption of both genes had no effects on tumor cell proliferation compared to untreated and LentiCas9-vector groups in both A549 and H2228 cells (Supplementary Figure S2A-S2C), illustrating the specific inhibition of A549 tumor cell proliferation by sgG12S targeting.

We further assessed the cell cycle of sgG12S- targeted A549 and H2228 cells (Figure 2D). The Cas9-sgG12S treated A549 cells were mostly arrested at S phase, and the ratio of the cell population at G2/M phase was downregulated correspondingly. There was no effect on the cell cycle of sgG12S-treated H2228 cells. Next, we examined the activities of the KRAS downstream signaling pathways, including the expression and activation of AKT and ERK (Figure 2E). The treatment of Cas9-sgG12S in A549 tumor cells dramatically suppressed the expression of the KRAS (G12S) protein, while the expression of wild- type KRAS protein in H2228 cells were not affected. In addition, the levels of phosphorylated-AKT (S473) and phosphorylated-ERK (T202/Y204) proteins were significantly downregulated in A549 cells edited with SpCas9-sgG12S, while another type of phosphorylated-ERK (T183/Y185) protein was not affected. As expected, AKT and ERK signaling pathways in H2228 cells were not affected by SpCas9-sgG12S. Collectively, our results suggested that the mutant allele-specific targeting by sgG12S can effectively inhibit tumor cell proliferation and arrest the cycle of tumor cells at S phase, likely through downregulation of the AKT and ERK signaling pathways.

### Transcription-repressing system dCas9-KRAB inhibited the proliferation of tumor cell lines *in vitro*

**Figure 3:**
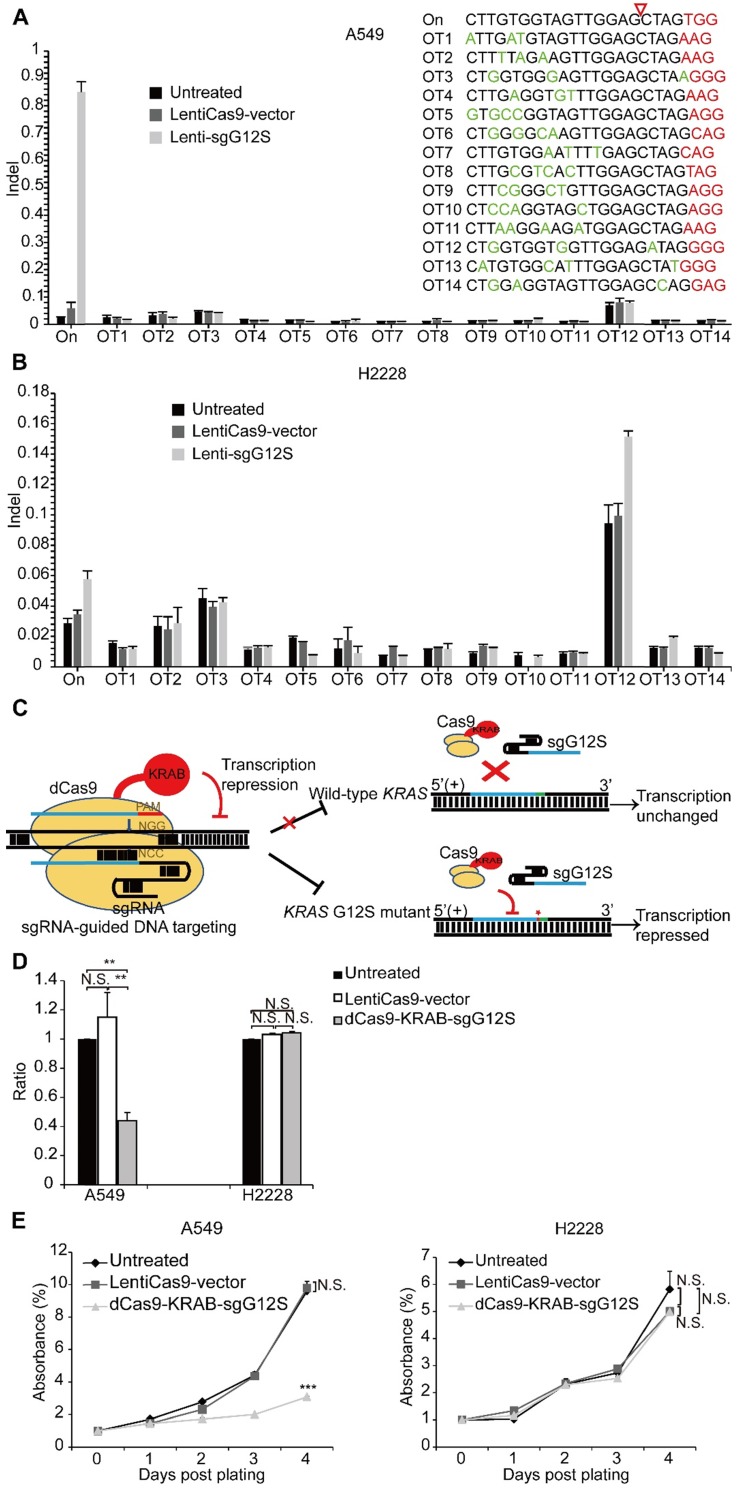
dCas9-KRAB mRNA-regulating system downregulated G12S transcription and inhibited tumor cell proliferation. (**A, B**) No off-target indels were noticeably caused by the CRISPR/Cas9 gene-cutting system at fourteen homologous sites that differed from the on-target sites by up to 4 nt. PAM sequences are shown in red and mismatched nucleotides are shown in green. On: on-target site. OT: off-target site. Cleavage position within the 20 bp target sequences is indicated by a red arrow. Error bar indicates S.E.M. (n=3 to 4). (**C**) Diagram of knocking down *KRAS* G12S mutant allele specifically by the dCas9-KRAB system. Blue strands: spacer; green strands: PAM sequence; red strands and star: single-nucleotide missense mutations. (**D**) qRT-PCR analysis of KRAS G12S mRNA expression. Error bars represent S.E.M. (∗) 0.01<P < 0.05, (∗∗) 0.001<P < 0.01, (∗∗∗) P < 0.001. (**E**) CCK-8 assay. Cell proliferation was determined at different timepoints by CCK-8 reagents. The relative number of cells of each group with different treatments was determined by normalizing the optical density at 490 nm of each CCK-8 reaction to the average optical density of the negative control groups.

We next explored whether there were off-target effects from the mutant allele-specific nuclease outside of the *KRAS* gene region by using targeted deep sequencing at 14 potential off-target sites (Supplementary Table S1). The potential off-target sites, which differ from the on-target site by up to a 4 nt-mismatch in the human genome, were identified by Feng Zhang lab's CAS-OFFinder algorithm (http://www.rgenome.net/cas-offinder/). No indel was detected at these sites in Cas9-sgG12S treated A549 and H2228 tumor cells (Figure 3A, 3B).

Genome-editing systems have the potential of causing undesirable double stranded breaks (DSB) in the genome (Figure 1B, 1D). In order to avoid the undesired disruption of the genome, we constructed a non-cutting transcription-regulating system, dCas9-KRAB system (Figure 3C), where KRAB is a transcriptional repressor to downregulate mRNA expression when binding to the regulatory elements of certain genes 25, 26. To test whether sgG12S linked to dCas9-KRAB may repress KRAS expression specifically in the G12S mutant allele, A549 and H2228 cells were infected by dCas9-KRAB-sgG12S and non-targeting control lentivirus. As expected, the transcription of the *KRAS* G12S mutant allele in dCas9-KRAB-sgG12S treated A549 cells was dramatically downregulated compared to the non-targeting control or untreated cells (Figure 3D), while in H2228 cells, the transcription of wild-type *KRAS* was not affected in all three groups. In addition, the effect on tumor cell growth was also investigated by a CCK-8 assay (Figure 3E). Consistently, the proliferation of dCas9-KRAB-sgG12S treated A549 cells was inhibited significantly in comparison to the controls, while no significant effect on H2228 tumor cell growth was observed. These results confirmed the *in vitro* specificity of the dCas9-KRAB system.

### Targeting KRAS-G12S mutant suppressed tumor growth in tumor-bearing mice

To further explore the effects of *KRAS*-sgG12S targeting *in vivo*, AdV-Cas9-sgG12S and non-targeting control adenovirus were constructed and packaged (Supplementary Figure S3A). Lentivirus has a relatively limited use for *in vitro* or *ex vivo* gene delivery due to their restricted insertional capacities and relatively low titers 29. Thus, the *in vivo* gene delivery experiments were conducted by adenoviral infection. The editing efficiency of AdVs was firstly confirmed in A549 and H2228 cells by a T7E1 assay (Supplementary Figure S3B) and sanger sequencing (Supplementary Figure S3C). As expected, AdV-Cas9-sgG12S specifically edited the *KRAS* G12S mutant allele in A549 cells, but not in H2228 cells harboring the wild-type *KRAS* gene. In addition, AdV-Cas9-sgG12S inhibited the proliferation of A549, but not H2228 tumor cells *in vitro* (Supplementary Figure S3D).

**Figure 4:**
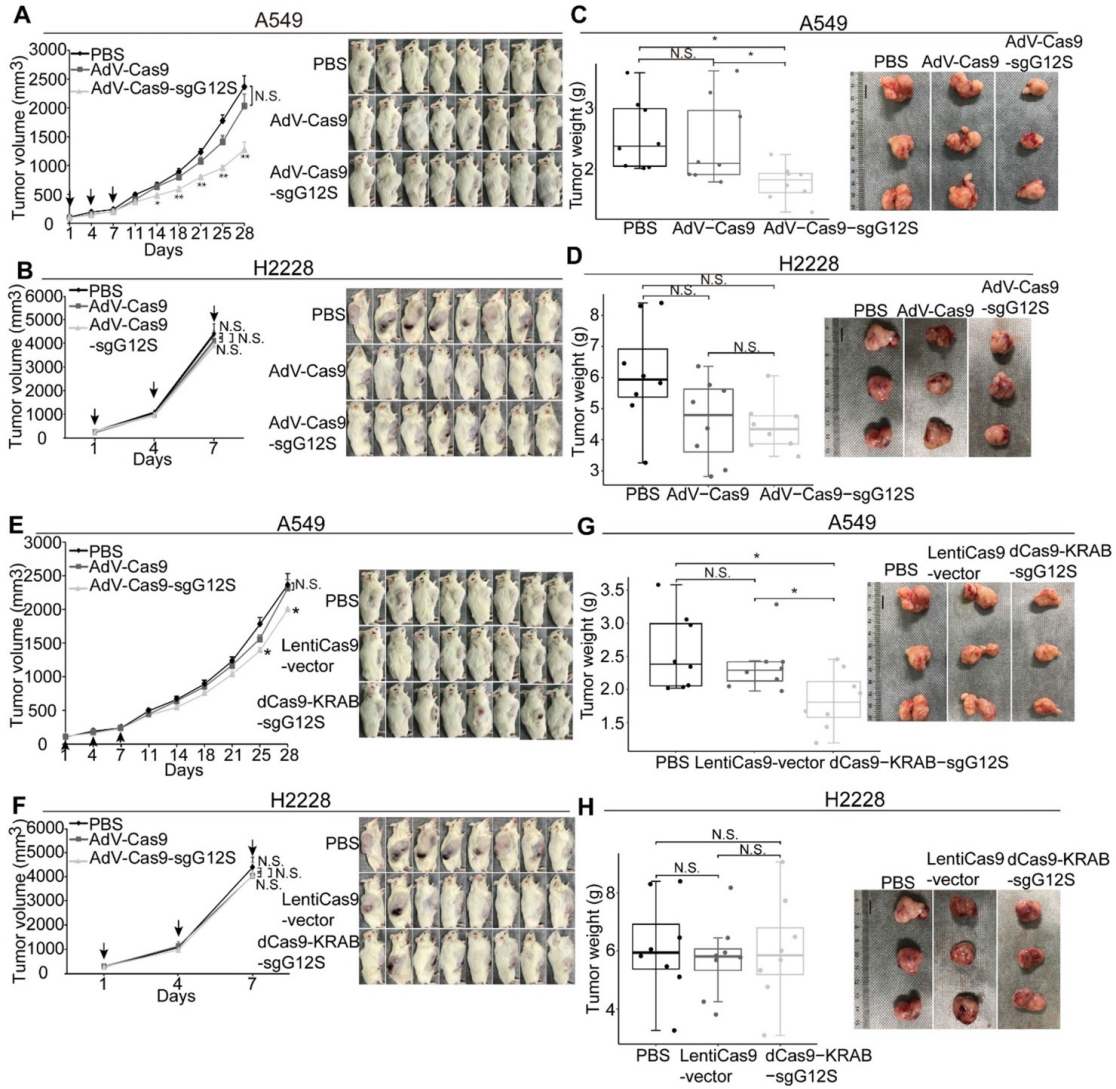
Antitumor effects of CRISPR-Cas9 and dCas9-KRAB systems in tumor xenograft models. Error bars represent SEM. 0.01<P < 0.05, (∗∗) 0.001<P < 0.01, (∗∗∗) P < 0.001. Values represent the mean ± S.E.M. (n=8 per group). (**A, B**) A549 and H2228 tumor-bearing mice were given intratumorally injections of PBS, AdV-Cas9, or AdV-Cas9-sgG12S adenoviruses on days 1, 4, and 7. Tumor growth was monitored twice a week post injection until the tumor volume exceeded 2000 mm^3^. (**C, D**) Weights of tumors removed from euthanized mice after 28 days in A549 tumor-bearing mice, and after 7 days in H2228 tumor-bearing mice. (**E, F**) A549 and H2228 tumor-bearing mice were intratumorally injected with PBS, LentiCas9-vector, or dCas9-KRAB-sgG12S lentiviruses on day 1, 4, and 7. Tumor growth was monitored twice a week post injection until the tumor volume exceeded 2000 mm^3^. (**G, H**) Weights of tumors removed from euthanized mice after 28 days in A549 tumor-bearing mice, and 7 days in H2228 tumor-bearing mice.

Next, we examined the effect of sgG12S editing in cell-derived xenograft models of A549 and H2228 cells (Figure 4A-D). Local injections of AdV-Cas9-sgG12S significantly inhibited tumor growth, resulting in a 46% reduction in tumor volume (P<0.01) in A549-bearing mice (Figure 4A). In contrast, tumor volumes of control groups treated with either PBS or AdV-Cas9 vector grew over time, reaching an average size of more than 2000 mm^3^ 28 days after treatment (Figure 4A). As expected, no significant difference in tumor volume was seen in AdV-Cas9-sgG12S, AdV-Cas9 vector, and PBS-treated mice implanted with H2228 cells containing the wild-type *KRAS* allele (Figure 4B). This confirmed the high specificity of *KRAS* G12S targeting *in vivo*. The tumor weight also significantly decreased by 30% in animals treated with AdV-Cas9-sgG12S, compared to control groups treated with either AdV-Cas9 vector or PBS (P<0.05) in A549 bearing mice (Figure 4C). Consistent with tumor volume, there was no difference in tumor weight of the H2228-implanted groups (Figure 4D).

To examine the efficacy of repressing G12S transcription by dCas9-KRAB system *in vivo*, NSG mice were xenografted with A549 and H2228 cells, and treated with dCas9-KRAB-sgG12S, non-targeting virus, or PBS once the tumor size reached a volume of 100-200 mm^3^ (Figure 4E-H). The mice xenografted with A549 cells and treated with dCas9-KRAB-sgG12S showed a 15.6% (P<0.05) decrease in tumor volume compared to a control (Figure 4E), and exhibited no notable metastasis or mortality during the observation period of 28 days. In contrast, the mice xenografted with H2228 cells treated with dCas9-KRAB-sgG12S did not show any inhibition of tumor growth and experienced a quick increase in tumor volume (Figure 4F). A similar rate of increase in tumor size was also observed in mice treated with the non-targeting vector or PBS. Tumor weights were measured in mice treated with different viral constructs (Figure 4G, 4H). A significant decrease in tumor weight (28.2%, P<0.05) was observed in dCas9-KRAB-sgG12S treated mice grafted with A549 cells (Figure 4G). In contrast, the H2228-bearing mice injected with either dCas9- KRAB-sgG12S, non-targeting vector or PBS treatment (Figure 4H) had little change in tumor weight.

Throughout the mouse studies of the gene- editing and transcription-repressing systems, no sign of weight loss (Supplementary Figure S4A-S4D) was observed. Taken together, these *in vivo* data suggested that gene targeting of mutant *KRAS* by SpCas9- sgG12S and dCas9-KRAB-sgG12S was effective and only restricted to the tumors with the *KRAS* mutations, with no obvious effects on the other cell types. In addition, the CRISPR/Cas9 genome- editing system targeting the mutant *KRAS* was more effective than the dCas9-KRAB mRNA-regulating system.

Potential off-target effects are an issue in CRISPR/Cas9 editing approaches. To analyze the off-target activity of CRISPR/Cas9 system, genomic DNA was isolated from mice treated with AdV-Cas9 and AdV-Cas9-sgG12S. Unintended mutations after CRISPR/Cas9 editing in mice AdV-Cas9-sgG12S #1, #2, and #3 were compared with mice AdV-Cas9 by whole exome sequencing (WES) (Table 2). There were some unique variants (Supplementary Figure S4E) and indels (Supplementary Figure S4F) in the control mice treated with AdV-Cas9, which suggests that the coverage of exome sequencing was not 100%. Likewise, 1, 4, and 2 indels were found only in mice AdV-Cas9-sgG12S #1, #2 and #3, respectively. In addition, a search in WES data was conducted for potential off-target sites for up to 4-nucleotide mismatches with on-target sites (Figure 3A). No off-target indels were identified in the comparison of the potential off-target sites with the indel locations identified by WES (Table 2).

Indels in mice treated with AdV-Cas9-sgG12S after background exclusion were listed in Table 3. *MED15*, a general transcriptional cofactor of the mediator complex involved in RNA polymerase II dependent transcription, was found to be edited in introns in all three tumor samples. In addition, *LOC105376360*, *JPH1,* and two other non-annotated gene loci were commonly found to be edited in mice AdV-Cas9-sgG12S #2 and #3. All indels were found with low abundance in the introns, except one insert that was found in the exon of *LOC286177* gene (Table 3). *LOC286177* is an RNA gene and affiliated with the lncRNA class. Considering there were no disorders found for the *LOC286177* gene, this genome editing method seems to be safe for manipulating genes *in vivo*.

### Disruption of KRAS-G12S significantly inhibited the protein expression of the mutant KRAS in tumor-bearing mice

**Figure 5:**
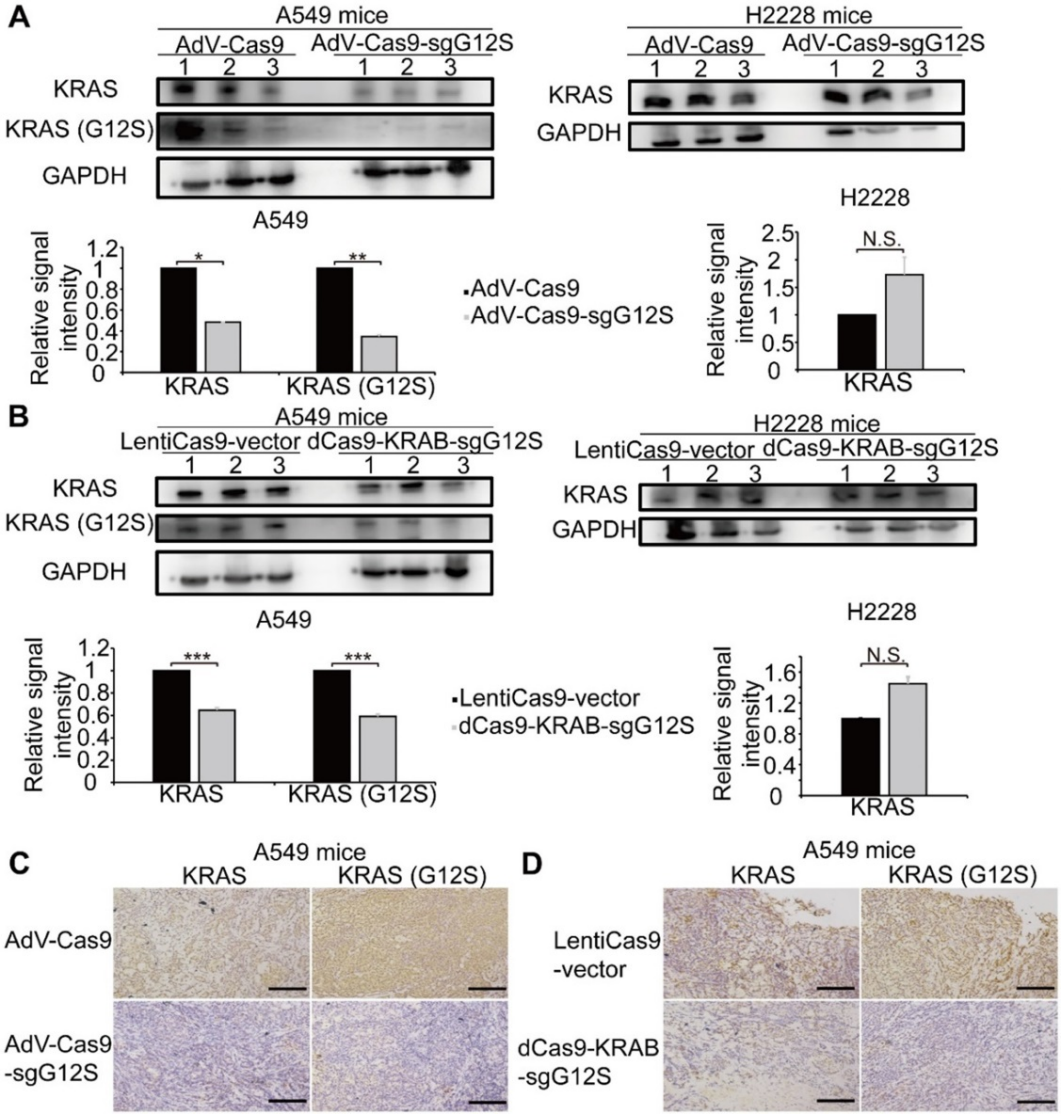
Targeting the *KRAS* G12S mutant allele significantly inhibited the expression of the KRAS mutant *in vivo*. Error bars represent SEM. (∗) 0.01< P < 0.05, (∗∗) 0.001< P < 0.01, (∗∗∗) P < 0.001. (**A**) Western blot analysis of the expression levels of total and mutant KRAS proteins in A549- and H2228-engrafted mice treated by the CRISPR-Cas9 gene-editing system, respectively. The optical density analysis was performed from the results of three replicate western blot samples. Tumors were removed from mice after 28 days in A549 tumor-bearing mice and after 7 days in H2228 tumor-bearing mice. (**B**) Western blot analysis of the expression levels in total and mutant KRAS proteins from A549- and H2228-engrafted mice treated by dCas9-KRAB mRNA-regulating system, respectively. The optical density analysis was performed from the results in three replicate samples. Tumors were removed from mice after 28 days in A549 tumor-bearing mice and after 7 days in H2228 tumor-bearing mice. (**C**) Immunohistochemical staining of KRAS and KRAS (G12S) were performed on tumor sections from A549 cells-engrafted mice treated with the CRISPR-Cas9 gene editing system. Scale bar: 100 µm. (**D**) Immunohistochemical staining of KRAS and KRAS (G12S) were performed on tumor sections from A549 cells-engrafted mice treated with the dCas9-KRAB system. Scale bar: 100 µm.

The antitumor efficacy of oncogenic mutant-specific gene editing and mRNA-regulating systems were further investigated by western blot and immunohistochemical (IHC) staining in the xenograft tumor tissues that had disrupted *KRAS*-G12S mutant alleles (Figure 5). A western blot (WB) assay revealed markedly reduced expression levels of KRAS and KRAS G12S mutant proteins in the tumor tissues of A549 cells-engrafted mice edited by AdV-Cas9-sgG12S, but not in the AdV-Cas9 treated control group. In the tumor tissues of H2228 cells-engrafted mice, the expression level of wild-type KRAS protein was not significantly changed in both the AdV-Cas9 or AdV-Cas9-sgG12S treated groups (Figure 5A). Consistently, dCas9-KRAB-sgG12S, but not LentiCas9-vector, treated tumor tissues exhibited markedly lower levels of both total and mutant KRAS proteins in A549-engrafted mice (Figure 5B). Importantly, tumor tissues from A549-engrafted mice treated with AdV-Cas9-sgG12S and dCas9-KRAB-sgG12S both showed significant reduction of KRAS G12S protein through in situ IHC staining, but this same reduction was not observed in the control groups (Figure 5C, 5D). This implied that the CRISPR/Cas9 system can effectively target and reduce KRAS mutant protein expression. Taken together, the data indicated that the application of both the gene-cutting CRISPR/Cas9 and mRNA-regulating dCas9-KRAB systems could lead to KRAS G12S protein downregulation *in vivo* and result in a strong anti-tumor efficacy.

### Extending the strategy of targeting a tumor-specific mutant locus with a gene editing system

**Figure 6:**
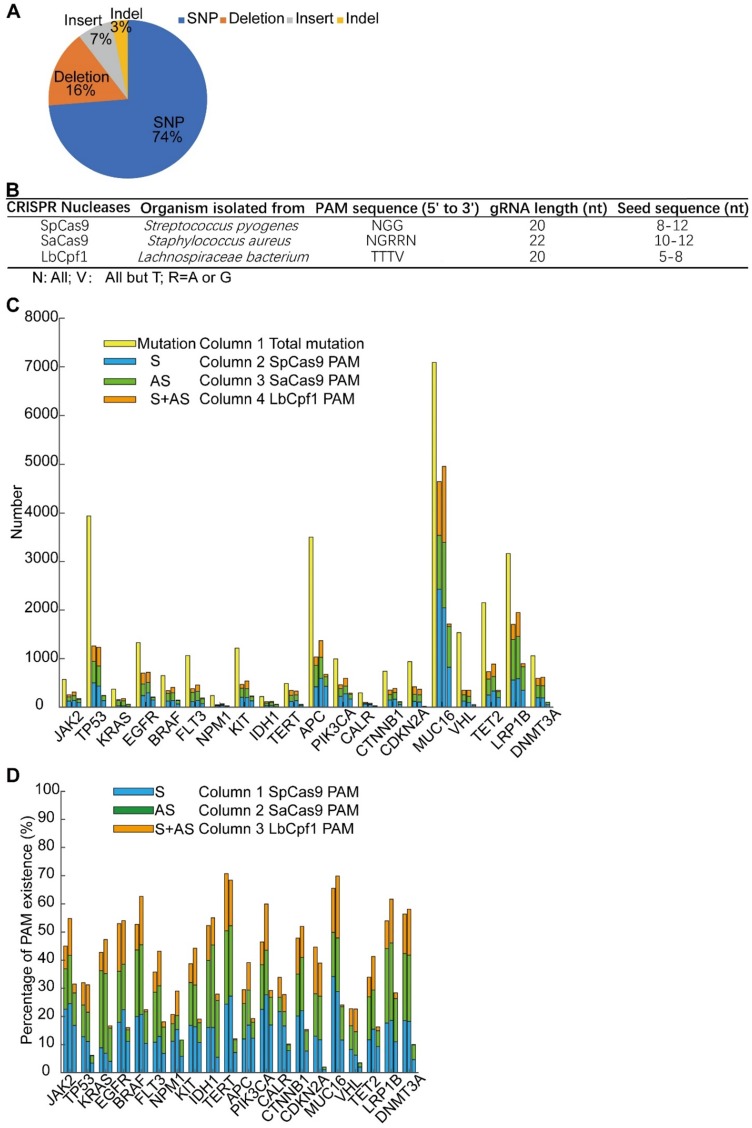
Screening of potential mutation-specific targets by CRISPR nucleases with bioinformatic analysis. (**A**) Percentage of different mutation types in the top 20 oncogenic genes. (**B**) Characteristics of three commonly used CRISPR nucleases: SpCas9, SaCas9, and LbCpf1. (**C**) Statistics of mutations that were in seed sequences or PAM sequences. S, sense strand. AS, anti-sense strand. (**D**) Percentage of 31555 SNV oncogenic mutations that could be targeted by CRISPR nucleases. S, sense strand. AS, anti-sense strand.

*The* Cas9-sgG12S editing system could be a highly specific strategy for targeting cancer driver gene mutations, with almost no difference in off-target effects between sgG12S and control groups (Figure 3A, 3B). Moreover, Cas9-sgG12S targeting specifically and efficiently inhibited tumor growth, both *in vitro* and *in vivo*. Thus, this approach holds great potential in treating *KRAS* G12S mutation-driven cancers. In order to extend this strategy to different DNA nucleases for targeting other oncogenic mutations, driver gene mutations were collected from the Cosmic database and the top 20 driver genes were selected to continue our proof-of-concept study (Supplementary Figure S5A). These high-frequency driver gene mutations, including *JAK2*, *TP53*, *KRAS*, *EGFR*, etc., are widely found in human malignancies 30 (Supplementary Figure S5B). Among these mutations, most of them are missense mutations, leading to single nucleotide variations (SNV) (Figure 6A). SNV occupies 74% of the overall mutations, while the percentage of deletions, insertions, and indels (insert and deletion) was 16%, 7%, and 3%, respectively.

There are a large number of mutations in each cancer driver gene, and it is important to identify whether these oncogenic mutations could be edited and which DNA nucleases could be applied for editing. To identify the mutations that could be specifically targeted by genome-editing nucleases including SpCas9, SaCas9, and LbCpf1 31, 32, we analyzed the SNV mutations to examine whether their flanking sequences fit the PAM or seed sequence requirements (Supplementary Figure S6). There was a length limitation of the seed sequence, and the seed sequence length of different nucleases differed (Figure 6B). In order to guarantee the targeting specificity, the lower limitation of the seed sequence length was used as the threshold in our analysis (Supplementary Figure S6). Among the 31555 SNV mutations of the 20 genes, about half of them can be edited by the above mentioned three CRISPR nucleases (Figure 6C). PAM sequences lying in the sense (S), the anti-sense (AS), or both sense and anti-sense (S+AS) sequences were counted. The genes carrying over 50% mutations that were able to be edited by either of the three CRISPR nucleases occupy half of the 20 genes, including *JAK2*, *EGFR*, *BRAF*, *IDH1*, *TERT*, *PIK3CA*, *CTNNB1*, *MUC16*, *LRP1B*, and *DNMT3A* (Figure 6D). The range of the SNV mutations that can be edited in each gene varies between 20.7% to 70.7%, and the highest predicted editing frequency was in the *TERT* gene by SpCas9. The distribution of the LbCpf1 PAM sequence was less frequent than that of SpCas9 and SaCas9. Altogether, specific targeting of cancer driver mutations by CRISPR nucleases has potential in treating oncogenic mutation-driven cancers, especially in the types of cancers that don't currently have effective therapies. Through bioinformatic analysis of 31555 SNV mutations, a reference list was generated to target these oncogenic mutations. The high-throughput bioinformatic pipeline could be used to analyze the distribution of PAM sequences and to estimate the target potential of other candidate genes.

## Discussion

The CRISPR/Cas9 genome-editing system is a powerful technique which can specifically target genomes or their mutated sequences. In our study, CRISPR/Cas9 was demonstrated to target the *KRAS* mutant allele, but not the wild-type allele. Other cancer-driven mutations, including the *EGFR* mutation (L858R), genomic rearrangements (*TMEM135-CCDC67* and *MAN2A1-FER* fusions), and the *BRAF* (V600E) driver mutation, have also been disrupted using CRISPR systems to control tumor growth 20, 21, 33. Unlike cancers driven by *KRAS* mutations, many EGFR inhibitors have been used in the treatment for lung cancers, including Erlotinib (Tarceva), Afatinib (Gilotrif), Gefitinib (Iressa), Osimertinib (Tagrisso), Dacomitinib (Vizimpro), and Necitumumab (Portrazza). There are also several clinical drugs that target cells with *BRAF* mutations, including Dabrafenib (Tafinlar) and Trametinib (Mekinist). Therefore, it is vital a system for targeting *KRAS* mutant alleles is developed, and may hold great promise for future cancer treatments.

In comparison to the traditional treatments using inhibitors for the KRAS pathway, the CRISPR/Cas9 system has extended the previous targeting from the protein level to the genomic DNA level, and this strategy can be widely and easily applied to other oncogenic mutations. The development of traditional inhibitors, including antibodies and small molecules, is complicated and the whole process is generally designed for a single target. For example, though the KRAS G12C inhibitors AMG 510, discovered by Amgen (NCT03600883), and MRTX849, invented by Mirati (NCT03785249), demonstrated promising clinical outcomes on their specific target, the G12C mutant, the two G12C inhibitors did not show any effect on other KRAS mutant alleles. Retargeting of different KRAS mutations at the protein level would be required to implement a new design, which is both time and cost-consuming. However, the CRISPR system is capable of targeting different mutant alleles specifically and precisely at the DNA level and can be used to target other oncogenic mutations by simply changing the sgRNA sequences. Combined with NGS, individual patients can be specifically treated with CRISPR/SpCas9 targeting their unique mutations. This editing of oncogenic mutations could be combined with inhibitors of KRAS or other oncogenic mutations, or immunotherapy, to further improve the anti-tumor efficacy.

In previous studies, the CRISPR/Cas9 system was harnessed to rectify disease-associated genetic defects 34-36 and deactivate disease-causing wild-type genes 37-39. However, the targeting was limited in specificity, and could not discriminate between the wild-type oncogenes and mutant alleles. Our study showed that a single-nucleotide mutation of a cancer driver gene can be selectively disrupted *in vitro* and *in vivo* by using sgRNAs, which can distinguish the mutant allele from the wild-type one. Among the four sgRNAs targeting mutations at the G12 locus, sgG12S showed the highest specificity with its ability to discriminate between the difference in a single-nucleotide polymorphism (SNP) (Figure 1B, D, E). To our best knowledge, this is the first report to demonstrate that the *KRAS* G12S mutant allele could be specifically targeted, thereby inhibiting tumor growth *in vivo*. Though Kim W. et al. 22 has targeted G12V, G12D, and G13D mutant alleles with lentiviral and adeno-associated viral (AAV) vectors, the mechanisms related to the tumor inhibition by targeting *KRAS* mutant alleles was not illustrated in their study. Zhao X. et al. 25 has used the CRISPR-Cas13a system to knockdown the KRAS G12D allele at the transcriptional level. The Cas13a system was reported to be tolerant to one mismatch and sensitive to two mismatches in the crRNA-target duplex. A second mismatch to the crRNA had to be introduced in their study, making their system more convoluted. In addition, the off-target effects of the study were not assessed. In our study, due to the limited availability of *KRAS* G12S cell lines, there was still the limitation of using only one single cell line for proof of concept. Therefore, two additional control sgRNAs, sgAAVS1 and sgTTN, were incorporated to rule out the possibility that A549 is uniquely sensitive to CRISPR editing of any type (Supplementary Figure S2A-S2C).

Our data showed that the disruption of the driver gene mutation in the *KRAS* G12S allele resulted in the inhibition of cancer cell growth *in vitro*. Moreover, on- and off-target indels, as well as cell cytotoxicity associated with CRISPR/Cas9 editing, were not detectable in H2228 cells with wild-type *KRAS* alleles. These results were consistent with *in vivo* data showing that tumor growth inhibition was not observed in AdV-Cas9-sgG12S treated H2228 tumors, demonstrating the specificity of CRISPR/ Cas9 for targeting a mutant allele. This finding was in line with the previous report by Cong et al. 19. In another study, CRISPR/Cas9 was used to target a mutant allele where the single nucleotide mutation generates a 5'-NGG-3' PAM sequence not present in the wild-type allele, thus enabling specific targeting of mutant alleles by the Cas9 nuclease 21. To extend this strategy to other cancer-driven mutations that are either located in seed sequences or to generate PAM sequences recognized by SpCas9 or other Cas9 variants, we chose the top 20 mutated genes and analyzed whether their mutations could be targeted by SpCas9, SaCas9, and LbCpf1 (Figure 6C, 6D, Supplementary Figure S6). Though some of these genes were reported as passenger genes like *MUC16*, most missense mutations were suspected of being driver mutations 40. We found that PAM sequences of CRISPR nucleases, especially for SpCas9 and SaCas9, are widely distributed around the mutated sites. These results indicate that this approach could be widely used to target other oncogenic mutations and be applied to other Cas9 families or variants. Furthermore, this approach could be utilized for multiple gene editing strategies in cancers frequently characterized by mutation heterogeneity, and to test functional relevance of tumor mutations using the CRISPR/Cas9 system 41, 42.

In contrast with two previous studies 22, 24, we assessed the off-target effects *in vitro* (Figure 3A, 3B) and *in vivo* (Table 2, Table 3 and Supplementary Figure S4E and S4F). Off-target indels were of low abundance and most of them were in introns, except one in an exon of an RNA gene, *LOC286177*. Considering no disorders were found for the *LOC286177* gene, it seems that this genome editing method is safe for manipulating genes *in vivo*. We further identified the associated mechanisms by which disruption of the *KRAS* G12S allele leads to the blockade of the AKT and ERK signaling pathways, thus inhibiting tumor growth. In addition, we assessed both the non-cutting transcription repression system, dCas9-KRAB, and the cleaving system, Cas9-sgG12S, to find that the transcription repression system is also capable of inhibiting tumor growth, but at a lower efficiency both* in vitro* and *in vivo*. Given that the dCas9-KRAB-sgG12S treatment only led to transient transcription repression by binding rather than by disrupting the genome sequence of the mutant gene, a constant growth inhibition in proliferating tumor cells may not be completely achieved using dCas9-KRAB-sgG12S. On the other hand, the genome-editing CRISPR/Cas9 system will be a more practical system to persistently abolish the oncogenic activation induced by the *KRAS* G12S mutant. Finally, among thousands of mutations of the top 20 cancer driver genes we surveyed, our bioinformatic analysis showed that more than 50% of the mutations in ten of the genes have the potential to be targeted by the CRISPR system. Due to the lack of PAM sequences, not every oncogenic mutation can be specifically targeted. Therefore, our bioinformatic pipeline could provide a convenient, efficient, and high-throughput method of predicting the editable sites.

Finally, though CRISPR/Cas9 systems provide powerful tools for fighting human diseases, undesired double stranded DNA breaks caused by off-target cutting of Cas9 nuclease are still its major concern in clinical application 43. There has been rapid progress in the field with the development of reagents that should increase editing efficiency and decrease off-target effects 44. Another obstacle for the translation of CRISPR/Cas9 is the inefficient delivery systems 45. CRISPR-based medicine was locally injected to subretinal for treating Leber congenital amaurosis (LCA10) (NCT03872479) 46. Lipid nanoparticle (LNP)-based delivery system has been developed by Intellia 47 (https://www.intelliatx.com/publications-and-presentations-2/). An IND application will be submitted for its LNP-based product, NTLA-2001, for the treatment of transthyretin amyloidosis (ATTR) (https://www.globenewswire.com/news-release/2019/10/31/1938637/0/en/Intellia-Therapeutics-Announces-Third-Quarter-2019-Financial-Results.html).

## Conclusions

We systematically demonstrated that gene-editing and mRNA-regulating systems specifically targeted the *KRAS* G12S mutant allele, which resulted in the inhibition of tumor cell proliferation and growth *in vitro* and *in vivo*. The findings demonstrate that these promising therapeutic alternatives could be applied for treating oncogenic mutation-driven cancers, though further hurdles need to be overcome. In addition, bioinformatic analysis of 31555 SNP oncogenic mutations could provide a potential pipeline for analyzing the distribution of PAM sequences for the screening of targeted genes.

## Materials and Methods

### Materials Cell lines and cell culture

HEK293T cells (ATCC, CRL-11268) were purchased from ATCC. HEK293T cells were cultured in DMEM (Gibco, 21063029) supplemented with 10% fetal bovine serum (Hyclone, SH30084.03HI), penicillin (100 IU/mL), and streptomycin (50 µg/mL). A549 (ATCC, CRM-CCL-185) and H2228 (ATCC, CRL-5935) cell lines were purchased from ATCC, USA. The cells were cultured in RPMI-1640 medium (Gibco, C22400500BT) supplemented with 10% fetal bovine serum (Hyclone, SH30084.03HI), penicillin (100 IU/mL), and streptomycin (50 µg/mL).

### Plasmid construction

pX330-U6-Chimeric vector (Addgene, 42230) and LentiCas9-vector plasmid with puromycin-resistance (Addgene, 52961) were purchased from Addgene. For sgRNA expression, oligonucleotides containing each target sequence were synthesized (BGI), followed by annealing in a thermocycler. Annealed oligonucleotides were ligated into the lentiCRISPR v2 plasmid digested with Bsm BI (Supplemental Figure S1).

### Lentivirus production

HEK293T cells were seeded at 70-80% confluency on 100 mm dishes. One day after seeding, the cells were transfected with a mixture (18 µg) of transfer plasmid (empty LentiCas9-vector or LentiCas9-vector containing sgRNA), psPAX2 (Addgene, 12260), and pMD2.G (Addgene, 12259) at a weight ratio of 4:3:2 using 54 µL PEI (Polysciences, 24765-1, 1 µg/µl). The medium was changed after 4-6 hours of incubation at 37 °C and 5% CO2. Viral supernatants were collected 72 hours after transfection and filtered through a 0.45 μm filter (Millipore, SLHP033RB), then ultra-centrifuged for 1.5 hours at 35,000 rpm (TYPE 45 Ti rotor of Beckman) at 4 °C to concentrate the virus. The resulting pellet was then resuspended in RPMI1640 medium without FBS, and stored at -80 °C. The lentiviral titers were determined with a Lenti-X™ qRT-PCR Titration Kit (Clontech, 631235).

### RNA extraction and qPCR

Total RNA was isolated from cells using TRIzol LS reagent (Invitrogen, 10296028) following the manufacturer's protocol. One microgram of RNA was then reverse transcribed using Primescript RT Reagent (Takara, RR047A). Quantitative PCR was performed using Fast Sybr Green Master mix (Thermo Fisher, 4385612) and the primers used were: *KRAS* forward, 5'-atgcatttttcttaagcgtcgatgg-3'; *KRAS* reverse, 5'-ccctgacatactcccaaggaaag-3'. Each messenger RNA (mRNA) level was measured as a fluorescent signal normalized based on the signal for GAPDH. Relative quantification was determined by the ΔΔCt method and normalized according to GAPDH.

### Cell proliferation assay and cell cycle analysis

Cells were seeded in 96-well plates at 1 × 10^3^ per well in 90 µL cell medium. Cell proliferation was assessed by Cell Counting Kit-8 (YEASEN, 40203ES80) according to the manufacturer's instructions. Briefly, 10 µL of CCK-8 solution was added to cell culture and incubated for 3-4 hours. Cell proliferation was evaluated by absorbance at 450 nm wavelength. For analysis of cell cycle, cells were plated in six-well plates at 6 × 10^5^ per well. After staining by propidium iodide (Sigma-Aldrich, P4170-10MG), the cell cycle distribution was analyzed by flow cytometry.

### Western blot analysis

A549 and H2228 cells were plated in six-well plates at a confluency of 70%. 48 hours after adenovirus infection, whole-cell extracts were prepared by lysing cells with the addition of 500 µL of hot SDS-PAGE buffer (Beyotime, P0015B). Tumor tissues were homogenized by TGrinder (Tiangen, OSE-Y30), and lysed with RIPA buffer containing complete protease inhibitor cocktail (Roche, 11697498001). Target proteins were detected by western blot analysis with the following antibodies: GAPDH mouse monoclonal antibody (Proteintech, 60004-1-Ig,), Akt (pan) (40D4) mouse monoclonal antibody (Cell Signaling Technology, 2920), Phospho-Akt (Ser473) (D9E) XP Rabbit mAb (Cell Signaling Technology, 4060), p44/42 MAPK (Erk1/2) (137F5) Rabbit mAb (Cell Signaling Technology, 4695), Phospho-p44/42 MAPK (Erk1/2) (Thr202/Tyr204) (D13.14.4E) (Cell Signaling Technology, 4370), mouse monoclonal Anti-MAP Kinase, activated (Diphosphorylated ERK-1&2) antibody (Sigma-Aldrich, M8159), Ras Antibody (Cell Signaling Technology, 3965), and Anti-RAS (G12S) Mouse Monoclonal Antibody (NewEast Biosciences, 26186).

### Generation, treatment and analysis of tumor xenografted mice

Xenograft mouse models of human lung cancer tumors were created by implanting A549 (5×106 cells in 200 µL DPBS (Gibco, C14190500BT)) or H2228 cells (2× 106 cells in 200 µL DPBS) through subcutaneous injections under the left upper limb in the abdomens of 6- to 8-week old male NCG mice. After tumor cell injection, when tumor volumes reached a range of 50-100 mm^3^, mice were randomly separated to one of five groups to receive PBS, AdV-Cas9, AdV-Cas9-sgG12S, LentiCas9-vector, or dCas9-KRAB-sgG12S (nine mice per group). The first day of treatment was designated as day 1. PBS, Adenovirus (1 × 10^9^ PFU in 10 µL DPBS), or lentivirus (5 × 10^10^ copies in 70 µL DPBS) was administered intratumorally on day 1, 4, and 7. Tumor growth inhibition was evaluated twice a week by measuring the length (L) and width (w) of the tumor. Tumor volume was determined using the following formula: volume = 0.523L(w)^2^.

### IHC staining

Tumor tissues were formalin-fixed, paraffin-embedded, and stained using anti-RAS (G12S) mouse monoclonal antibody (NewEast Biosciences, 26186), followed by incubation with the HRP-conjugated corresponding secondary antibody (Millipore, AP160P). The expression levels were evaluated by the H-score method. Scoring was independently reviewed in parallel by two experienced pathologists.

### PAM analysis

*G*enomic variants were annotated and prioritized based on previous reports 47. ANNOVAR 48 was used to annotate the COSMIC v88 mutation database (perl table_annovar.pl humandb/hg19_cosmic88.txt humandb/ -buildver hg19 -out cosmic -remove -protocol refGene -operation gx -nastring . -csvout), and to select the variants located in the exons of the 20 cancer driver genes. Based on the gene mutation and wild-type genome information (https://www.ncbi.nlm.nih.gov/assembly/GCF_000001405.25), we applied Pandas (https://pandas.pydata.org/), a python package, to analyze the COSMIC SNP mutation information to generate a data frame. We applied Pyfaidx 49, a python package to extract specific sequences from the GRCh37.p13 reference genome. PAM sequences of SpCas9, SaCas9, and LbCpf1 CRISPR nucleases were analyzed in the GRCh37.p13 reference genome. Once the SNP mutations were in the seed region of the PAM sequences, we considered it to be editable by CRISPR nucleases.

### Statistical analysis

Significance of all data was determined using two-tailed Student's t-test, and p-values <0.05 were considered statistically significant.

### Data availability

Data supporting this study has been deposited in the CNSA (https://db.cngb.org/cnsa/) of CNGBdb with accession code CNP0000672, and submitted to the NCBI (PRJNA576375), available online: https://www.ncbi.nlm.nih.gov/bioproject/576375.

### Ethics approval and consent to participate

The mouse model studies were performed according to the guidelines provided by the Chinese Animal Welfare Act and approved by the Institutional Review Board on Bioethics and Biosafety of BGI.

## Supplementary Material

Supplementary figures and table.

## Figures and Tables

**Table 1:** Occurrence of *KRAS* G12S mutation in different diseases

Diseases	Occurrence of *KRAS* G12S (%)
Rectal Carcinoma	2.56
Colorectal Adenocarcinoma	1.84
Colorectal Carcinoma	1.66
Non-Small Cell Lung Carcinoma	0.5
Squamous Cell Lung Carcinoma	0.23
Myelodysplastic Syndromes	0.19
Acute Myeloid Leukemia	0.14

**Table 2:** Variants in mice identified using WES

Mouse sample	AdV-Cas9	AdV-Cas9-sgG12S#1	AdV-Cas9-sgG12S#2	AdV-Cas9-sgG12S#3
All variants	88	81	258	243
All indels	12	9	19	16
Possible off-target sites after alignment with predicted off-target sites	N/A	0	0	0

N/A, not applicable

**Table 3:** Distribution and classification of indels in AdV-Cas9-sgG12S treated mice

Sample	Gene	Reference	Alteration	Abundance	Exon/intron	Reframed
**AdV-Cas9-sgG12S#1**	MED15	TGTG	TGTGGTG	1	intron	No
	ZMAT4	GA	GATA	1	intron	No
**AdV-Cas9-sgG12S#2**	LOC105376360	AG	AGTGGAGGGGTATCTCG	9	intron	No
	MED15	TGTG	TGTGGTG	3	intron	No
	Non-annotated	ACCC	ACCCC	7	N/A	N/A
	RAB22A	CGGGGGG	CGGGGGGG	6	intron	No
	Non-annotated	C	CCG	1	N/A	N/A
	JPH1	TCCCC	TCCCCCC	2	intron	No
	Non-annotated	CAT	C	5	N/A	N/A
	CPQ	C	CGCCG	1	intron	No
	Non-annotated	AACAACAACAA	AACAACAA	2	N/A	N/A
**AdV-Cas9-sgG12S#3**	LOC105376360	AG	AGTGGAGGGGTATCTCG	7	intron	No
	MED15	TGTG	TGTGGTG	4	intron	No
	Non-annotated	CCC	CCCCCGCC	1	N/A	N/A
	Non-annotated	ACCC	ACCCC	5	N/A	N/A
	LOC286177	TGGGGG	TGGGGGG	3	exon	Yes
	JPH1	TCCCC	TCCCCCC	5	intron	No
	Non-annotated	CAT	C	6	N/A	N/A

## Abbreviations

PDACs: pancreatic ductal adenocarcinomas
CRCs: colorectal adenocarcinomas
LACs: lung adenocarcinomas
CRISPR: Clustered regularly interspaced short palindromic repeats
SpCas9: *S. pyogenes* CRISPR associated protein 9
EGFR: epidermal growth factor receptor
dCas9: dead Cas9
KRAB: Krüppel associated box
PAM: protospacer adjacent motif
NGS: next generation sequencing
CFA: colony formation assay
DSB: double stand break
IHC: immunohistochemical
WB: western blot
SNV: single nucleotide variation
SNP: single- nucleotide polymorphism
AAV: adeno-associated viral
ATCC: American Type Culture Collection
DMEM: Dulbecco's modified Eagle's medium
GAPDH: glyceraldehyde 3-phosphate dehydrogenase
H&E: hematoxylin and eosin
