# Supplementary material for: Selective targeting of the oncogenic KRAS G12S mutant allele by CRISPR/Cas9 induces efficient tumor regression
Source: Theranostics. 2020 Apr 6;10(11):5137–53. doi: 10.7150/thno.42325 (PMC7163449; doi:10.7150/thno.42325)
Supplement: Supplementary file 1 — Supplementary figures and table. [file thnov10p5137s1.pdf]

Figure S1

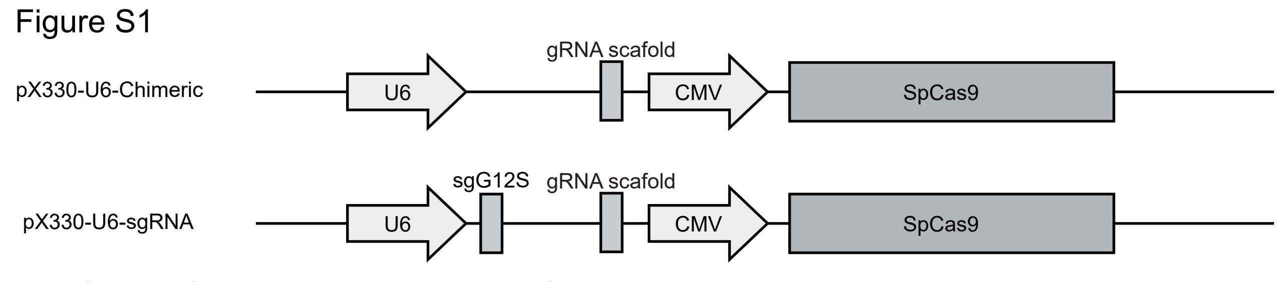

Figure S1. Maps of pX330 vectors, including pX330-U6-Chimeric blank vector and pX330-U6-sgRNA expressing vector.

Figure S2

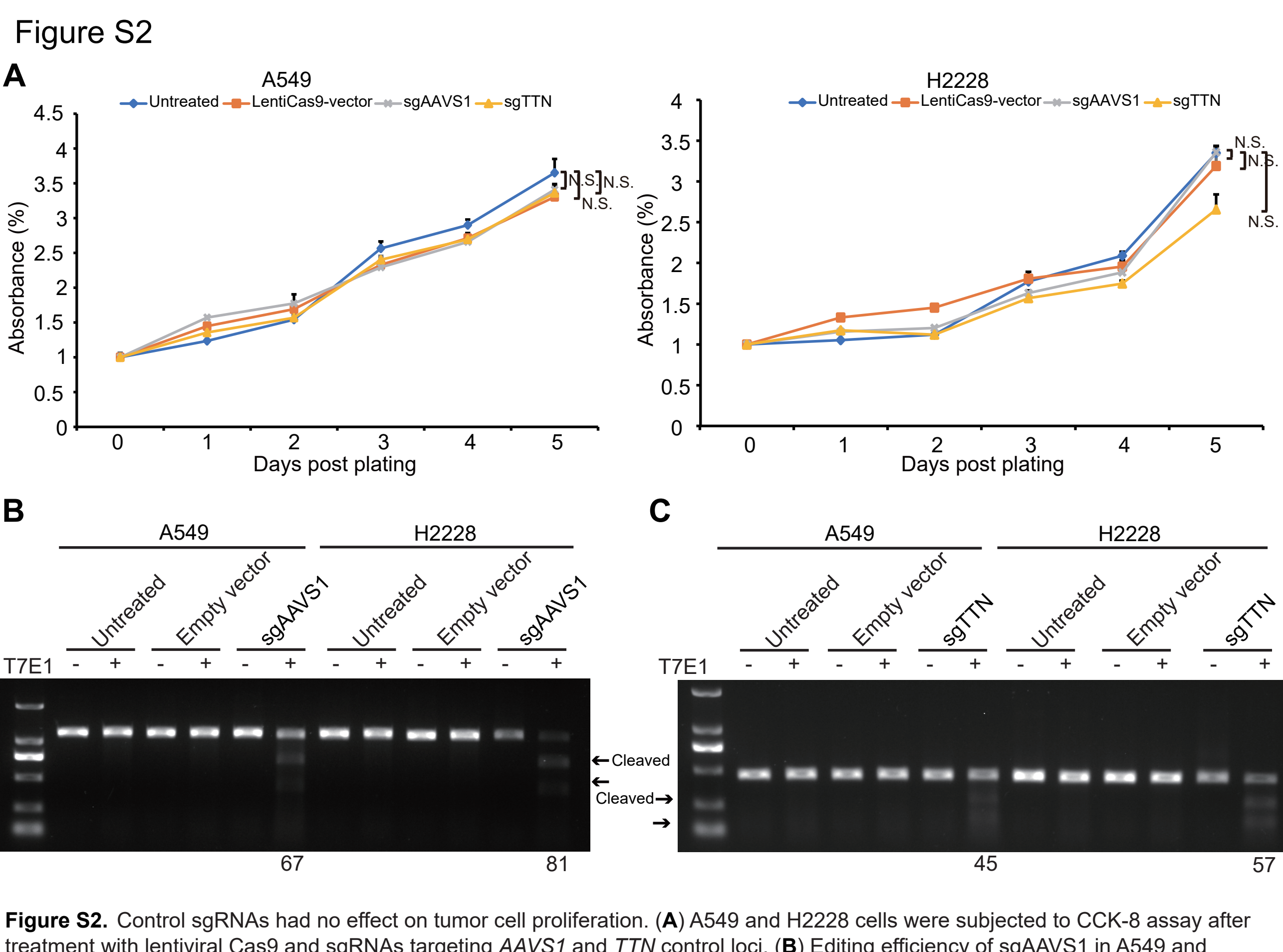

Figure S2. Control sgRNAs had no effect on tumor cell proliferation. (**A**) A549 and H2228 cells were subjected to CCK-8 assay after treatment with lentiviral Cas9 and sgRNAs targeting *AAVS1* and *TTN* control loci. (**B**) Editing efficiency of sgAAVS1 in A549 and H2228 cells. Effective editing of genes is presenting by the appearance of cleaved band. And the gene editing efficiency is listed in lanes correspondingly. (**C**) Editing efficiency of sgTTN in A549 and H2228 cells. Effective editing of genes is presenting by the appearance of cleaved band. And the gene editing efficiency is listed in lanes correspondingly.

Figure S3

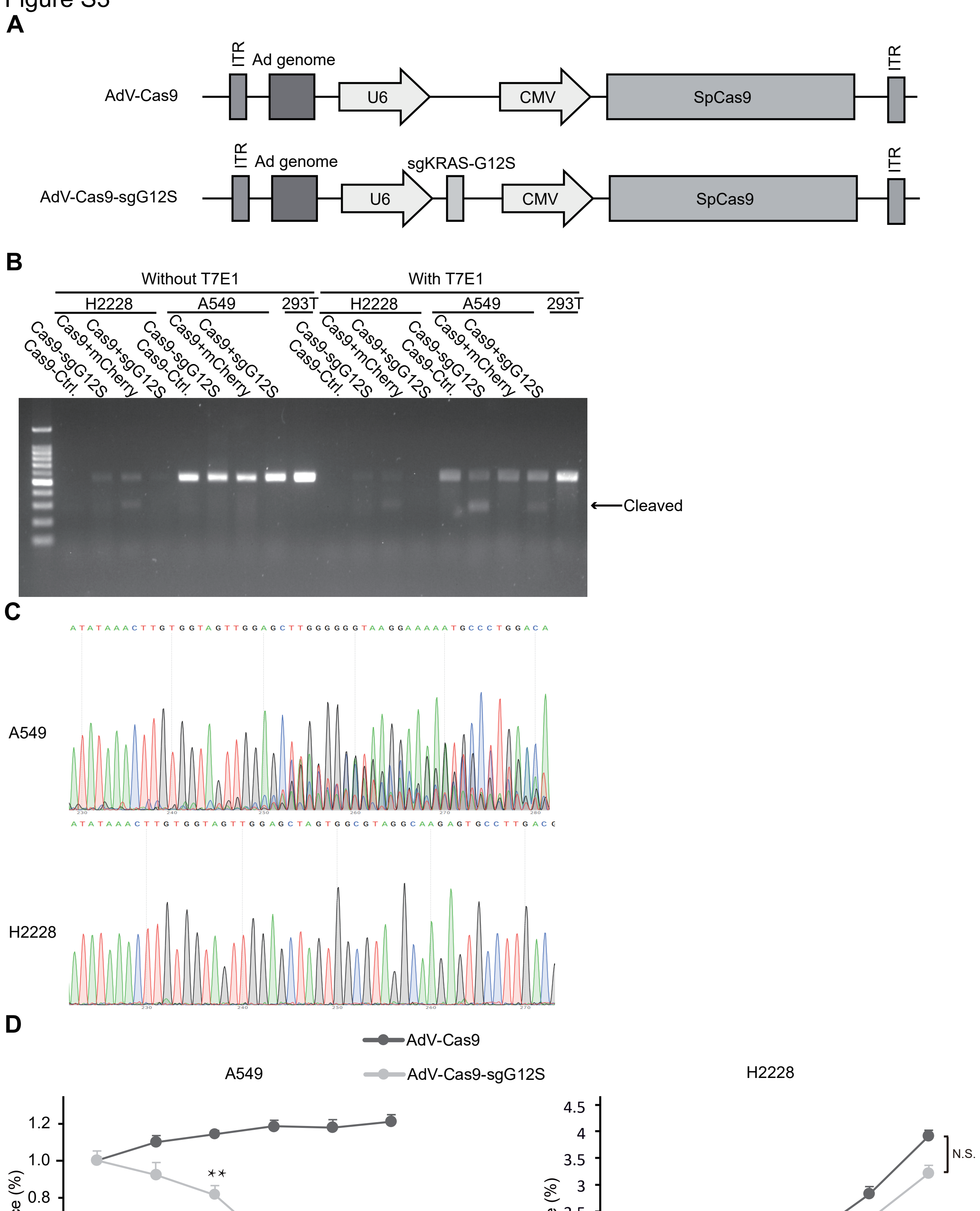

Figure S3. Editing efficiency and inhibition of tumor cells of AdV-Cas9-sgG12S adenovirus. (**A**) Maps of adenoviral vectors, including AdV-Cas9 blank vector and sgG12S guide RNA expressing vector AdV-Cas9-sgG12S. (**B**) Gene editing efficiency and specificity of AdV-Cas9-sgG12S adenovirus were confirmed by sanger sequencing in A549 and H2228 cells. (**C**) Gene editing efficiency and specificity of AdV-Cas9-sgG12S adenovirus were confirmed by sanger sequencing in A549 and H2228 cells. (**D**) CCK-8 assay. Cell proliferation was accessed by using CCK-8 reagents at different timepoints after plating. The number of cells in cultures with different treatments was determined by the optical density at 490 nm.

Figure S4

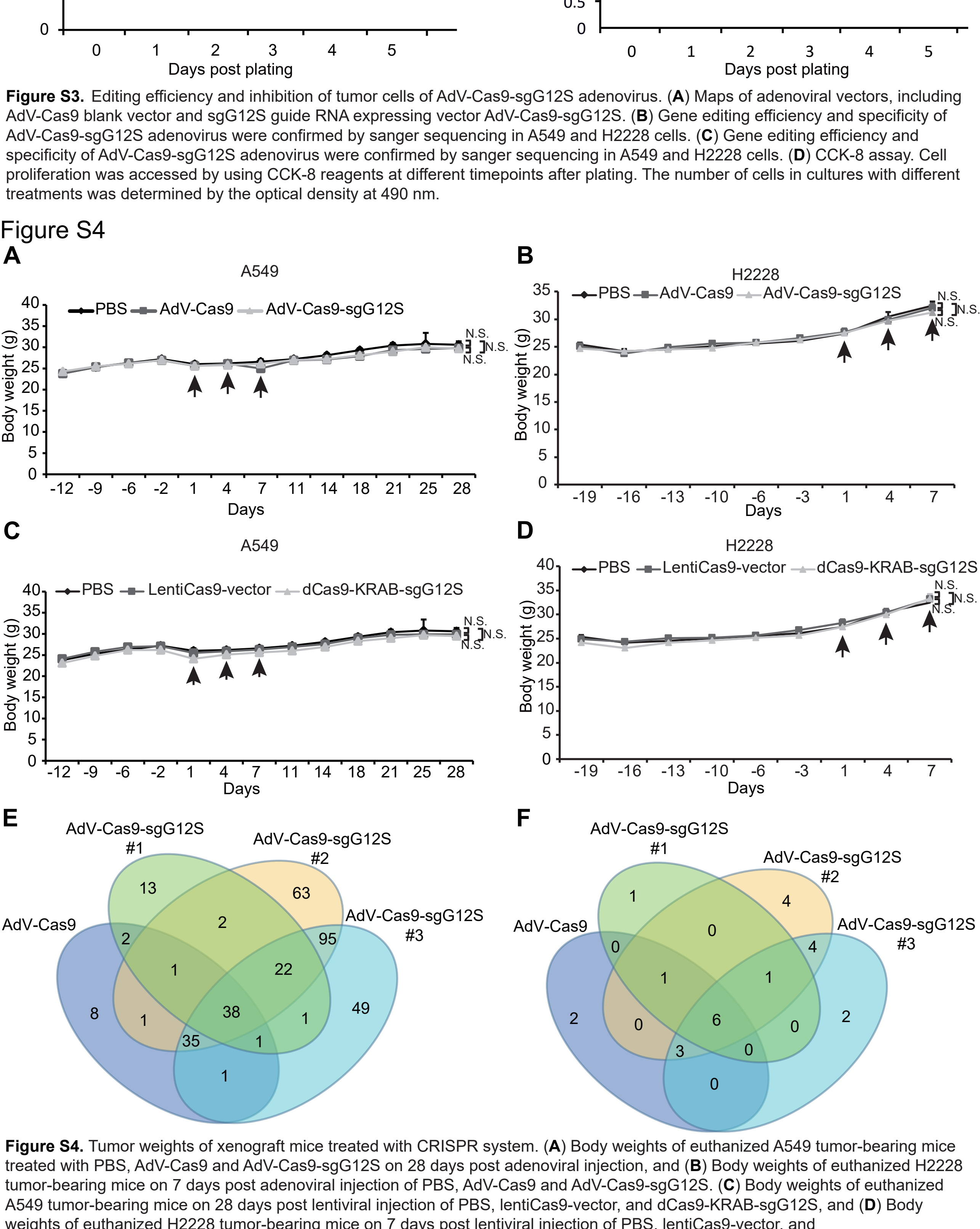

Figure S4. Tumor weights of xenograft mice treated with CRISPR system. (**A**) Body weights of euthanized A549 tumor-bearing mice treated with PBS, AdV-Cas9 and AdV-Cas9-sgG12S on 28 days post adenoviral injection, and (**B**) Body weights of euthanized H2228 tumor-bearing mice on 7 days post adenoviral injection of PBS, AdV-Cas9 and AdV-Cas9-sgG12S. (**C**) Body weights of euthanized A549 tumor-bearing mice on 28 days post lentiviral injection of PBS, lentiCas9-vector, and dCas9-KRAB-sgG12S, and (**D**) Body weights of euthanized H2228 tumor-bearing mice on 7 days post lentiviral injection of PBS, lentiCas9-vector, and dCas9-KRAB-sgG12S. (**E**) A Venn diagram showing the number of all variants in mice treated with AdV-Cas9 and AdV-Cas9-sgG12S. (**F**) A Venn diagram showing the number of indels in mice treated with AdV-Cas9 and AdV-Cas9-sgG12S.

Figure S5

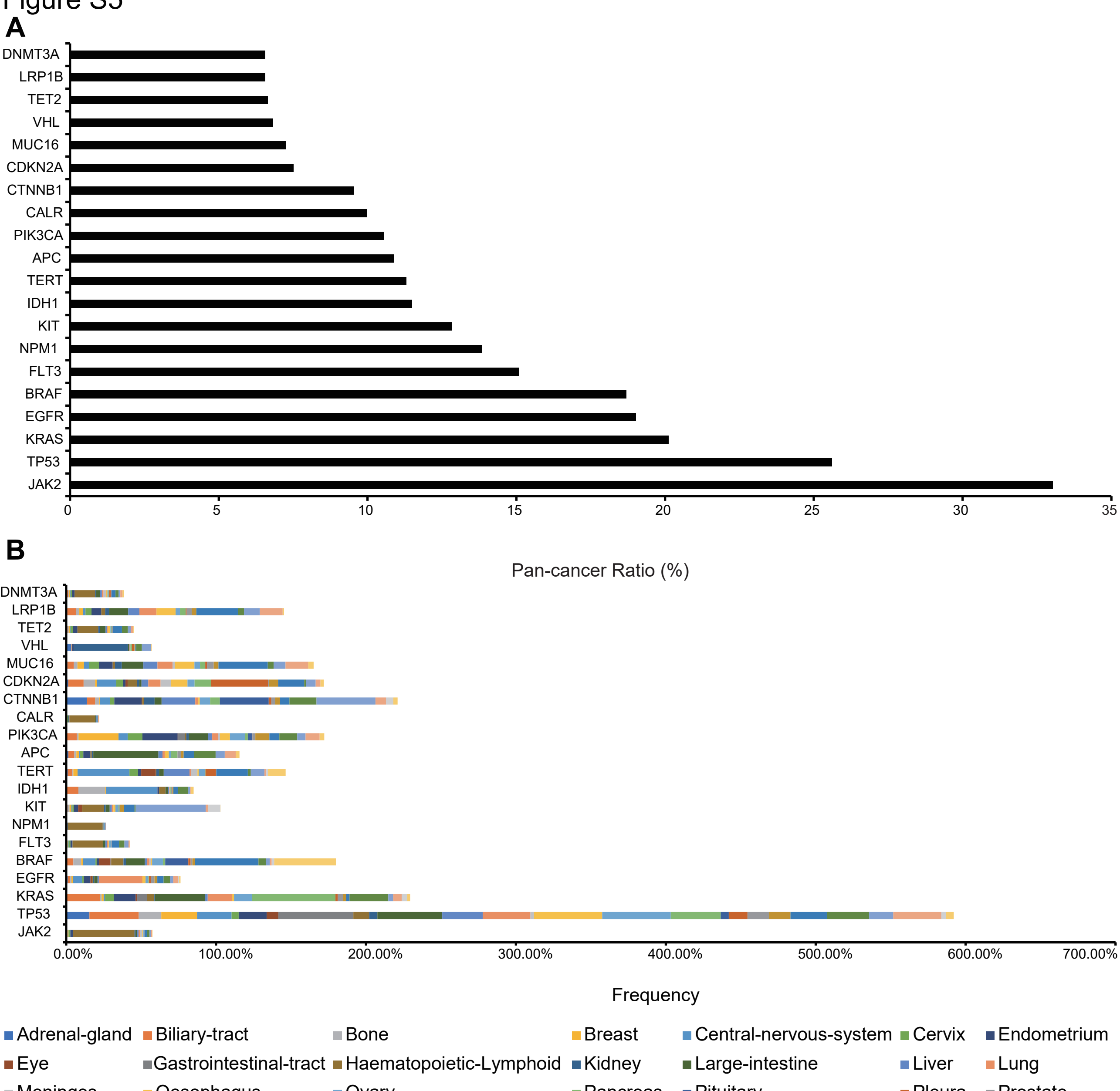

Figure S5. Frequency and distribution of top 20 human cancer driver gene mutations. (**A**) Top 20 oncogenic mutations discovered from Cosmic database. (**B**) Distribution of oncogenic mutations in human tissues.

Figure S6

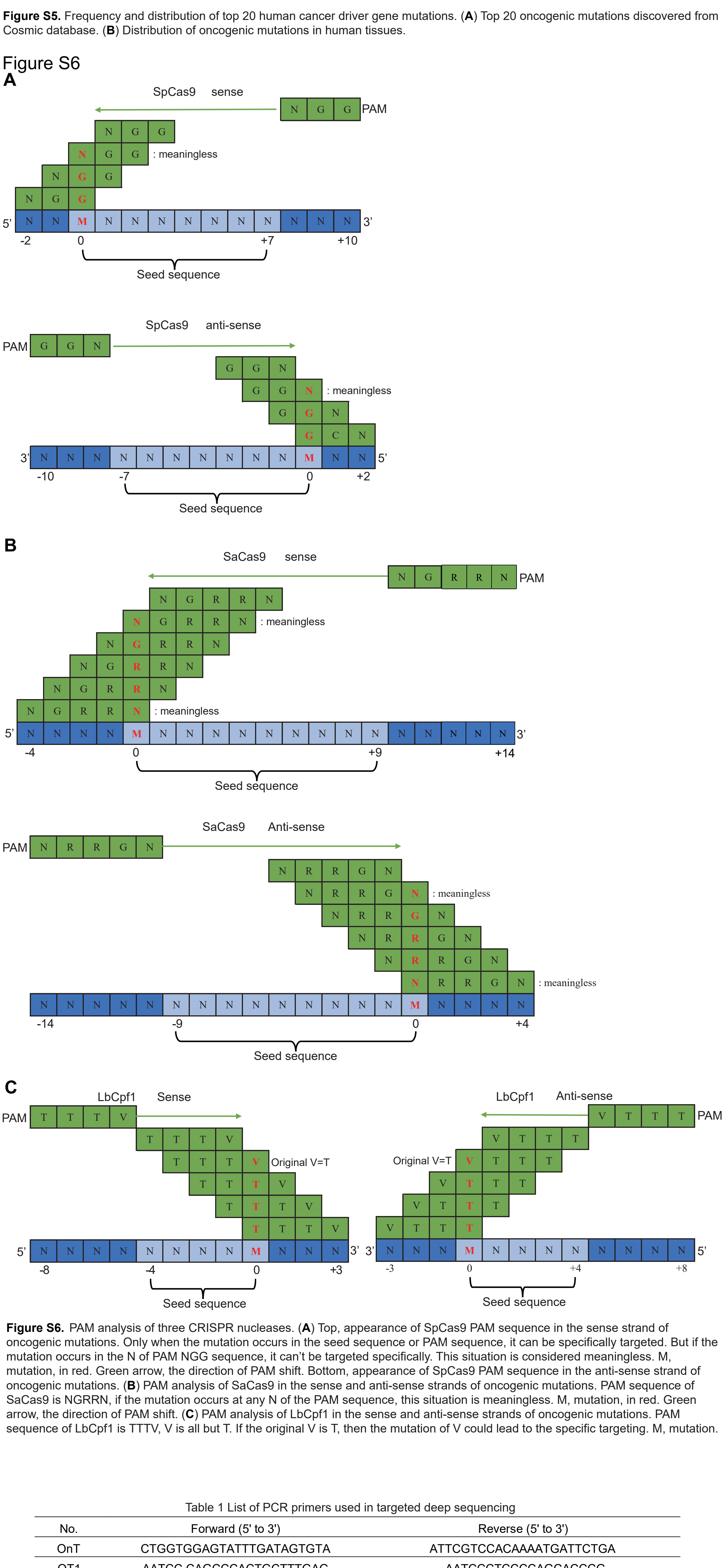

Figure S6. PAM analysis of three CRISPR nucleases. (**A**) Top, appearance of SpCas9 PAM sequence in the sense strand of oncogenic mutations. Only when the mutation occurs in the seed sequence or PAM sequence, it can be specifically targeted. But if the mutation occurs in the N of PAM NGG sequence, it can't be targeted specifically. This situation is considered meaningless. M, mutation, in red. Green arrow, the direction of PAM shift. Bottom, appearance of SpCas9 PAM sequence in the anti-sense strand of oncogenic mutations. (**B**) PAM analysis of SaCas9 in the sense and anti-sense strands of oncogenic mutations. PAM sequence of SaCas9 is NGRRN, if the mutation occurs at any N of the PAM sequence, this situation is meaningless. M, mutation, in red. Green arrow, the direction of PAM shift. (**C**) PAM analysis of LbCpf1 in the sense and anti-sense strands of oncogenic mutations. PAM sequence of LbCpf1 is TTTV, V is all but T. If the original V is T, then the mutation of V could lead to the specific targeting. M, mutation.

Table 1 List of PCR primers used in targeted deep sequencing

No.	Forward (5' to 3')	Reverse (5' to 3')
OnT	CTGGTGGAGTATTTGATAGTGTA	ATTCGTCCACAAAATGATTCTGA
OT1	AATCC CAGCCCACTGCTTTGAG	AATCCCTCCCCAGCACCCG
OT2	TGGTTATGTTTCCTTTTTGACTGC	CCCTGGAGATTCTGATACAGTGGA
OT3	GCACAGAAGAACAGCAGCGAGGTAG	CCATAAAAAATCTCATCAGCCCCAA
OT4	TTGTCTCTGCCCTTATGGATTGC	CCAAGAGAAAAGCATTTGTCCTGAG
OT5	GCAGGATGTAGATGTGGGTAAGG	ATTAGGTGGTAAAGGTCGTGGGAA
OT6	CAGGTGAAAGAAATCGTTAGGGACA	ACAAACAGTTCCTGGCACACG
OT7	AGGTTATGTGGCTTATTCAAGGTCA	GTCGTAACCTTCTATGTGACTATTG
OT8	CCTGAAGAGATGGGTGTATTTTGG	TAAGCCTTTCTACCTCCTGGGG
OT9	CCACAAAGAAATGAGAAACAGT	TAACCCCTAAGTATTATCCCAGAA
OT10	GTCACACAGTCAGTGGCAGAGAAGA	ACAGTACAGGCACAGGGTGGCAGC
OT11	GTGAGGCAAGGAAGTTTGATTTT	TTCTCTATCTCCAGTCTCTGCTTT
OT12	GGATAAGAGCACTTGGGCAGA	AACTGTAGGAAATAAGCAAAGGAGA
OT13	CACACAAAATCCATAAAATCGGTCA	GCTACAAGAATGGTACACCCAGTCG
OT14	CTGAACTAAAGAAGGAACTGCCGCC	ACTCCCGTCGTTCGGCTCGGTCCT
